# Diabetes and Hypertension Risk Across Acculturation and Education Levels in Hispanic/Latino Adults: The Hispanic Community Health Study/Study of Latinos

**DOI:** 10.1001/jamahealthforum.2025.0273

**Published:** 2025-03-28

**Authors:** Eliseo J. Pérez-Stable, Asmi Panigrahi, Saida I. Coreas, Erik J. Rodriquez, Aimee Afable, Tali Elfassy, Carmen R. Isasi, Jeffrey S. Gonzalez, Martha L. Daviglus, Amanda Hinerman, Aida Giachello, Krista M. Perreira, Linda C. Gallo

**Affiliations:** 1 Division of Intramural Research, National Heart, Lung, and Blood Institute, Bethesda, Maryland; 2 School of Public Health, SUNY Downstate Health Sciences University, Brooklyn, New York; 3 Department of Medicine, University of Miami, Miami, Florida; 4 Department of Epidemiology & Population Health, Albert Einstein College of Medicine, Bronx, New York; 5 Ferkauf Graduate School of Psychology, Yeshiva University, Bronx, New York; 6 Department of Epidemiology & Population Health, Albert Einstein College of Medicine, Bronx, New York; 7 Institute for Minority Health Research, University of Illinois at Chicago; 8 Division of Intramural Research, National Heart, Lung, and Blood Institute, Bethesda, Maryland; 9 Department of Preventive Medicine, Feinberg School of Medicine, Northwestern University at Chicago, Chicago, Illinois; 10 Department of Social Medicine, School of Medicine, University of North Carolina, Chapel Hill; 11 Department of Psychology, San Diego State University, Chula Vista, California

## Abstract

**Importance:**

Acculturation among Hispanic/Latino populations, defined as adaptation to US lifestyle and culture, is often assumed to lead to adverse health outcomes that will reduce the immigrant health advantage.

**Objective:**

To evaluate the risks for incident diabetes and hypertension by levels of acculturation and educational attainment.

**Design, Setting, and Participants:**

This study used data from the Hispanic Community Health Study/Study of Latinos, a population-based cohort study of men and women aged 18 to 74 years who identified as Central American, Cuban, Dominican, Mexican, Puerto Rican, or South American living in 4 urban locales in the US. Visit 1 spanned March 2008 to June 2011; visit 2 spanned October 2014 to December 2017.

**Main Outcomes and Measures:**

Multivariable logistic regression was used to evaluate the association between language acculturation level defined by the Short Acculturation Scale for Hispanics (SASH) and educational attainment (less than high school graduate vs more than high school) and incident diabetes and hypertension by heritage group.

**Results:**

Of 11 623 adult participants, 1207 (10.4%) were of Central American heritage, 1645 (14.2%) of Cuban heritage, 1021 (8.8%) of Dominican heritage, 11 623 (41.3%) of Mexican heritage, 1801 (15.5%) of Puerto Rican heritage, and 795 (6.8%) of South American heritage. The mean (SE) age of all participants was 43.1 (0.3) years, and 7345 (56.3%) were female. A total of 8697 (71.4%) were born outside the US, 4358 (32.5%) had less than a high school education, and 7475 (58.3%) were less acculturated (SASH score less than 2). Incident rates of diabetes (total cohort, 14.6% [95% CI, 13.6%-15.6%]) and hypertension (total cohort, 20.4% [95% CI, 19.0%-21.9%]) varied across heritages; Mexican individuals (17.2% [95% CI, 15.5%-19.0%]) had the highest diabetes incidence and Dominican individuals the highest hypertension incidence (27.1% [95% CI, 22.7%-31.4%]). Persons with more educational attainment were at lower risk for diabetes and hypertension independent of acculturation, and more acculturated participants had a lower risk for incident diabetes. Less acculturated with lower socioeconomic status (SES) were more likely to have incident diabetes (weighted predicted probability [WPP], 0.17 [95% CI, 0.14-0.19]), and more acculturated with lower SES had the highest predicted probability of hypertension (WPP, 0.19 [95% CI, 0.15-0.23]). More acculturated with higher SES were at lower risk of diabetes (WPP, 0.11 [95% CI, 0.09-0.13]) and had a lower predicted probability of hypertension (WPP, 0.10 [95% CI, 0.08-0.12]).

**Conclusions and Relevance:**

In this cohort study, the association of acculturation with health outcomes is not unidirectional and interacts with educational attainment in determining incident diabetes and hypertension. The Hispanic and Latino paradox, in which immigrants have a health advantage, is influenced by more factors than acculturation and may persist with higher educational attainment. These observations may inform prevention and treatment strategies associated with cardiometabolic health in Hispanic/Latino populations.

## Key Points

**Question:**

Does increased acculturation among Hispanic/Latino populations lead to adverse health outcomes?

**Findings:**

In this cohort study of 11 623 Hispanic/Latino adults from the Hispanic Community Health Study/Study of Latinos, the risks of incident diabetes and hypertension varied across 5 heritages; Mexican individuals (17.2%) had the highest diabetes incidence, and Dominican individuals, the highest hypertension incidence (27.1%). Persons with higher educational attainment were at lower risk of diabetes and hypertension, independent of acculturation, and more acculturated participants had a lower risk for incident diabetes.

**Meaning:**

The Hispanic/Latino immigrant paradox in which immigrants have better health outcomes is influenced by more factors than acculturation, and the immigrant health advantage may persist with higher educational attainment.

## Teaser

This cohort study evaluates the risks for incident diabetes and hypertension among Hispanic/Latino adults in 4 US cities by levels of acculturation and educational attainment.

## Introduction

Hispanic/Latino individuals are the largest racial and/or ethnic minority population in the US, and in 2020, nearly 70% of these individuals were born in the US.^1^ Hispanic/Latino communities face unique health challenges, including substantially higher prevalence of diabetes, obesity, and uncontrolled hypertension, as well as higher death rates from diabetes.^2,3,4,5^ Despite these disparities, Hispanic/Latino adults have a lower prevalence of cardiovascular disease morbidity and mortality and a longer life expectancy than White individuals in the US.^6,7^ Previous research has suggested that immigrant health outcomes are associated with time spent in the US or with adaptation to US lifestyle and culture, also known as acculturation.^8,9^

This health advantage of Hispanic/Latino persons has been described as an epidemiological paradox because a socially and economically disadvantaged population has better health outcomes than expected. These diverging health patterns may be due to the “healthy immigrant effect,”^10^ and there is evidence from recent mortality analyses that US-born Hispanic/Latino individuals have a shorter life expectancy.^11^ However, the underlying explanation for these diverging patterns of health outcomes remains unknown and warrants further research.^12^ The predominant paradigm has been that Hispanic/Latino immigrants arrive in the US with an initial health advantage that erodes over time and across successive immigrant generations. This paradigm points to the acculturation process as a potential mechanism influencing health outcomes. Although researchers have been discussing this phenomenon for decades,^10,13,14^ there remains a paucity of empirical evidence investigating the role of acculturation and social and economic disadvantage on cardiometabolic health in the Hispanic/Latino paradox.

The construct of acculturation is usually measured by language preference, language-based scales, or years spent in the US, and being more acculturated has been suggested to adversely affect health outcomes among Hispanic/Latino individuals.^8,9^ In theory, the process of acculturation leads to adopting behaviors of the host country population, which may subsequently lead to losing the immigrant health advantage and ultimately higher prevalence of chronic diseases. However, there is limited evidence to support this assumption, and most data are based on Mexican heritage populations. This is particularly relevant for diabetes and hypertension, which have been inconsistently associated with acculturation levels with results in both decreased and increased risk directions.^3,4,15,16,17,18,19,20,21^ Much of the existing research on immigrant health emphasizes the multifaceted and dynamic nature of the immigrant experience, making it especially important to consider other factors that may shape how acculturation influences Hispanic/Latino health behaviors and outcomes.^22^

The US Hispanic/Latino population comes from many Latin American countries, even though the majority of the research on acculturation evaluates Hispanic/Latino populations in aggregate or focuses exclusively on those of Mexican heritage.^23,24^ Given that diabetes and hypertension prevalence differ by heritage among Hispanic/Latino populations,^2,25,26,27^ it is possible that the seemingly inconsistent association between acculturation and cardiometabolic disease is moderated by other factors related to the diverse immigrant experience. Other studies suggest a key moderating factor may be socioeconomic status (SES),^18^ which is independently associated with cardiovascular risk factors,^2^ diabetes,^28^ and hypertension^14,29^ and has also been shown to vary by heritage.^2,3,4,5,30^

The theoretical framework known as the segmented assimilation model has been proposed to explain how SES may interact with acculturation to explain varying health outcomes among immigrant populations.^31^ This framework may help explain the seemingly inconsistent relationships between acculturation and diabetes/hypertension, as well as the Hispanic/Latino paradox.^31^ The segmented assimilation model proposes that there are multiple dimensions of the acculturative experience based on how immigrants experience social mobility in the US, changes in SES across successive generations, and the evolution of how receptive the host country is to immigrants. For example, while greater acculturation levels may lead to an increased risk of cardiometabolic disease for some immigrants, others may be protected from such an increased risk due to the preservation of culture-related health behaviors provided by a greater SES. To date, research on how acculturation and SES together are associated with health outcomes among Hispanic/Latino populations is limited.^13,17,18,21^ Analyses rooted in segmented assimilation theory from the Hispanic Established Populations for Epidemiologic Studies of the Elderly and the Sacramento Area Latino Study on Aging suggested that SES moderates the association between acculturation and diabetes risk.^17,18^

Understanding the social and behavioral mechanisms of cardiometabolic disease within the framework of segmented assimilation could influence both prevention and treatment strategies and subsequently have a beneficial impact on future disease rates. This study aimed to identify acculturation and socioeconomic factors that are associated with the risks of diabetes and hypertension among Hispanic/Latino individuals from different heritages, within the framework of segmented assimilation, using data from the Hispanic Community Health Study/Study of Latinos (HCHS/SOL). Our goals were to (1) evaluate the association of increased acculturation with incident diabetes and hypertension after considering socioeconomic factors and (2) compare these results by self-reported heritage in HCHS/SOL. We hypothesized that there would be significant variation in the extent of cardiometabolic disease risk across segmented groups, which were based on differences in levels of both acculturation and SES.

## Methods

### Data Source and Sampling

The HCHS/SOL is a community-based cohort study of 16 415 self-identified Hispanic/Latino individuals at visit 1, aged between 18 and 74 years, enrolled through 4 field centers (Bronx, New York; Chicago, Illinois; Miami, Florida; and San Diego, California). The goal of HCHS/SOL was to describe the prevalence of risk and protective factors for cardiovascular disease, diabetes, and pulmonary disease and to quantify fatal and nonfatal events from these conditions and all-cause mortality.^32^ Individuals self-identified their national heritage as Central American, Cuban, Dominican, Mexican, Puerto Rican, South American, other, or more than 1 heritage. Sampling weights were generated to reflect the probabilities of selection at each stage due to participants being selected with unequal probabilities. Further details on the sample design, cohort selection, and study rationale have been published previously.^32,33^

Visit 1 spanned March 2008 to June 2011, and visit 2 spanned October 2014 to December 2017. Individuals that completed visit 2 were included in this analysis. Sampling weights were recalculated after visit 2 to account for nonresponse, and thus the analysis applies to the HCHS/SOL study population and is not biased. Institutional review board review and informed consent were not required for this analysis of deidentified data, according to the National Institutes of Health policy and 45 CFR §46. We followed the Strengthening the Reporting of Observational Studies in Epidemiology (STROBE) reporting guideline.

### Measures of Acculturation and SES

Acculturation level was assessed at visit 1 using the Short Acculturation Scale for Hispanics (SASH) language subscale.^34^ A continuous SASH language score was calculated using responses on a 5-point Likert scale (1 [only Spanish] to 5 [only English]) which consisted of the following 6 items: (1) language(s) in which the person reads and speaks, (2) language(s) used as a child, (3) language(s) usually spoken at home, (4) language(s) in which the person usually thinks, (5) language(s) the person usually speaks with friends, and (6) language(s) preferred for movies, television, and radio programs, with a higher summary score reflecting a higher level of acculturation. Acculturation level was categorized as a dichotomous variable at visit 1 with values from 1 to less than 2 defined as less acculturated and values of 2 to 5 as more acculturated based on an observed bimodal distribution of acculturation in the population.

Self-reported educational attainment at visit 1 was used as the measure of SES. The highest level of education achieved was measured and categorized as no high school diploma or equivalent, high school diploma or equivalent, and some college or more. A high school diploma or equivalent or less was categorized as lower SES and some college (greater than high school education) as higher SES.

Additional measures of acculturation and socioeconomic factors assessed at visit 1 were considered in the sensitivity analysis. The acculturation measures were years living in the US (<10 years vs ≥10 years), language preference, and a question on Hispanic ethnic identity. SES measures included annual household income (<$30 000 vs ≥$30 000), home ownership, and perceived SES on a ladder scale of 1 to 10. As a sensitivity analysis, these additional measures of acculturation and SES were analyzed (eTables 2 and 3 in Supplement 1).

### Diabetes and Hypertension

Diabetes was defined based on the American Diabetes Association definition and determined by serum glucose levels adjusted for fasting time, post–oral glucose tolerance test glucose levels (if available), glycosylated hemoglobin A_1C_, and self-reported diabetes at visit 1 and visit 2. Hypertension was defined using the National Health and Nutrition Examination Survey definition of a measured systolic blood pressure of 140 mm Hg or higher and/or diastolic blood pressure of 90 mm Hg or higher and/or currently taking antihypertensive medications at visit 1 and visit 2. Family history of diabetes and hypertension was assessed based on a previous physician diagnosis of diabetes or hypertension for the participant’s parents or siblings. Incidence of hypertension and diabetes in the HCHS/SOL cohort were previously published.^26,27^

### Demographic Characteristics and Other Variables

Age, sex, and birthplace were assessed at visit 1. Birthplace was determined by self-reported country of birth. US-born participants included those born in the 50 US states; Washington, DC; or US territories, including Puerto Rico. All other participants were categorized as born outside the US. Other factors measured at visit 1 were health insurance status, poor diet quality, physical activity, and heritage group. Education, age, health insurance status, and obesity (body mass index ≥30 [calculated as weight in kilograms divided by height in meters squared]) were measured at visits 1 and 2.

### Statistical Analysis

Descriptive characteristics, age-standardized to the 2010 US Census population, were computed for the overall population and presented stratified by heritage. The 2 levels of acculturation and 2 levels of SES defined the 4 categories of segmented groups: (1) less acculturated and lower SES, (2) less acculturated and higher SES, (3) more acculturated and lower SES, and (4) more acculturated and higher SES. Segmented groups were justified by statistically significant interaction terms between acculturation and SES. Frequencies of prevalent diabetes and hypertension were calculated for each heritage group and each segmented group at visits 1 and 2. Incident diabetes and hypertension were defined at visit 2 as new diagnoses absent in visit 1.

We used multivariable logistic regression to evaluate the association between our main variable of interest (segmented group) and incident diabetes and hypertension for the total sample and by heritage. Models included all participants at visit 2 with nonmissing data for variables related to demographics, acculturation, SES, diabetes outcomes, and hypertension outcomes at both visits. Models controlled for visit 1 age, gender, health insurance status, study site, family history of diabetes and hypertension, obesity, poor diet, physical activity, and birthplace. Multivariate models are shown by Central American, Cuban, Dominican, Mexican, Puerto Rican, or South American heritage groups. Age-standardized prevalence and incidence of diabetes and hypertension and predicted probabilities were not calculated for the South American group due to the heterogeneous backgrounds from up to 13 different countries.

As a sensitivity analysis, models were repeated for control variables available at visit 2, including education, age, health insurance status, and obesity. If a variable was unavailable at visit 2, the visit 1 value was carried forward. We report predicted probabilities, calculated using marginal standardization, for the results of these models. In accordance with segmented assimilation theory, we separately assessed whether years in the US (<10 years vs ≥10 years) would modify via stratification our modeling results. Survey data analysis procedures for means, percentages, and regression modeling were used to account for the complex sampling and weighting procedures, as well as item nonresponse. Although 29.2% of the sample from visit 1 was missing data at visit 2, sampling weights were recalculated to account for nonresponse and missing data at visit 2. Analyses were conducted using SAS, version 9.4 (SAS Institute, Inc). The analysis took place from March 2021 to December 2024.

## Results

Of 11 623 adult participants, 1207 (10.4%) were of Central American heritage, 1645 (14.2%) of Cuban heritage, 1021 (8.8%) of Dominican heritage, 11 623 (41.3%) of Mexican heritage, 1801 (15.5%) of Puerto Rican heritage, and 795 (6.8%) of South American heritage. The mean (SE) age of all participants was 43.1 (0.3) years, and 7345 (56.3%) were female (Table 1).

**Table 1.** Weighted, Age-Adjusted Demographic Characteristics of Hispanic/Latino Individuals by National Heritage at Visit 1: Hispanic Community Health Study/Study of Latinos^a^

Characteristic	No. (%)^b^						
	Total (N = 11 623)^d^	Heritage^c^					
		Central American (n = 1207)	Cuban (n = 1645)	Dominican (n = 1021)	Mexican (n = 4806)	Puerto Rican (n = 1801)	South American (n = 795)
Age, mean (SE), y	43.1 (0.3)	41.7 (0.6)	49.2 (0.6)	41.9 (0.7)	40.1 (0.4)	45.2 (0.6)	44.2 (0.9)
Age group, y							
18-29	1459 (21.7)	159 (23.0)	115 (11.5)	144 (24.1)	684 (26.0)	185 (19.2)	74 (16.5)
30-39	1538 (19.7)	203 (24.4)	142 (13.5)	114 (18.0)	749 (24.1)	168 (14.9)	116 (21.9)
40-49	3123 (24.1)	317 (22.8)	456 (25.4)	291 (26.6)	1327 (23.7)	425 (23.5)	219 (23.7)
50-59	3407 (18.7)	366 (18.0)	525 (21.9)	294 (18.2)	1310 (15.9)	596 (23.0)	238 (22.9)
60-74	2090 (15.8)	162 (11.8)	406 (27.8)	177 (13.2)	733 (10.4)	427 (19.3)	147 (15.0)
Sex							
Female	7345 (56.3)	793 (59.3)	925 (52.1)	713 (64.7)	3117 (57.0)	1101 (53.8)	497 (58.5)
Male	4278 (43.7)	414 (40.7)	720 (47.9)	308 (35.3)	1689 (43.0)	700 (46.2)	298 (41.5)
Birthplace							
Born outside the US	8697 (71.4)	1155 (93.8)	1573 (90.6)	958 (90.1)	4066 (79.4)	43 (2.4)	762 (93.6)
Born in US states, districts, or territories	2896 (28.6)	52 (6.2)	72 (9.4)	63 (9.9)	739 (20.6)	1758 (97.6)	33 (6.4)
Acculturation SASH scale (range, 1-5)^e^							
Less acculturated (<2)	7475 (58.3)	946 (72.9)	1389 (74.8)	741 (65.0)	3202 (59.9)	514 (22.2)	574 (63.3)
More acculturated (≥2)	4097 (41.7)	255 (27.1)	252 (25.2)	280 (35.0)	1595 (40.1)	1281 (77.8)	215 (36.7)
SASH language, mean (SE)	2.0 (0.02)	1.7 (0.1)	1.6 (0.1)	1.8 (0.04)	2.0 (0.03)	3.1 (0.1)	1.8 (0.04)
Frequency	11 572	1201	1641	1021	4797	1795	789
Education level							
Less than high school	4358 (32.5)	490 (39.2)	376 (20.5)	432 (39.8)	2101 (37.5)	690 (36.4)	189 (20.4)
High school graduate/equivalent	2900 (26.8)	264 (22.8)	461 (28.7)	209 (21.5)	1236 (27.7)	476 (26.8)	192 (26.4)
Greater than high school/equivalent	4322 (40.7)	451 (38.0)	808 (50.8)	380 (38.6)	1460 (34.8)	634 (36.8)	414 (53.2)
Household income							
<$30 000	7476 (66.0)	820 (72.4)	1131 (71.4)	721 (73.8)	2937 (60.1)	1165 (66.9)	521 (62.7)
≥$30 000	3512 (34.0)	288 (27.6)	362 (28.6)	239 (26.2)	1687 (39.9)	557 (33.1)	253 (37.3)
Health insurance status							
Any insurance	5868 (51.9)	398 (34.5)	631 (40.7)	742 (74.2)	2160 (45.5)	1465 (81.2)	291 (42.3)
No insurance	5589 (48.1)	798 (65.5)	1001 (59.3)	239 (25.8)	2623 (54.5)	298 (18.8)	489 (57.7)
Segmented groups^f^							
Less acculturated, lower SES	5170 (38.7)	644 (51.5)	726 (38.4)	525 (46.3)	2510 (45.2)	405 (17.1)	308 (34.4)
Less acculturated, higher SES	2286 (19.7)	300 (21.4)	663 (36.4)	216 (18.6)	685 (14.7)	109 (5.1)	266 (28.9)
More acculturated, lower SES	2067 (20.6)	106 (10.5)	110 (10.8)	116 (15.0)	820 (20.0)	757 (46.1)	70 (12.8)
More acculturated, higher SES	2024 (21.1)	149 (16.6)	142 (14.4)	164 (20.0)	773 (20.1)	523 (31.8)	145 (23.9)

^a^ Analyses were weighted to account for complex sampling and study design of the Hispanic Community Health Study/Study of Latinos and age-adjusted to the 2010 US Census population and restricted to participants that completed visit 1 and visit 2. Visit 1 spanned 2007 to 2011.

^b^ Percentages are based on nonmissing values.

^c^ *P* < .001 for all comparisons.

^d^ A total of 348 individuals did not identify with a specific heritage or self-reported other heritage; results are included in the total group but not shown as a separate heritage.

^e^ A continuous Short Acculturation Scale for Hispanics (SASH) language score was calculated using responses on a 5-point Likert scale (1 [only Spanish] to 5 [only English]) which consisted of the following 6 items: (1) language(s) in which the person reads and speaks, (2) language(s) used as a child, (3) language(s) usually spoken at home, (4) language(s) in which the person usually thinks, (5) language(s) the person usually speaks with friends, and (6) language(s) preferred for movies, television, and radio programs. The average of the 6 questions indicates the degree of language acculturation, with a higher number reflecting greater acculturation.

^f^ Less vs more acculturation categories were determined by 2-level acculturation SASH variable. Lower vs higher socioeconomic status (SES) was determined by using educational attainment as a proxy measure, high school level or less categorized as low SES, and some college or more as high SES.

The mean (SE) ages of Cuban (49.2 [0.6] years), Puerto Rican (45.2 [0.6] years), and South American individuals (44.2 [0.9] years) tended to be older when compared with Mexican (40.1 [0.4] years), Dominican (41.9 [0.7] years), and Central American individuals (41.7 [0.6] years). All heritages had greater proportions of women than men. A total of 8697 participants (71.4%) were born outside the 50 US states; Washington, DC; or the US territories in the overall cohort, although 1758 of 1801 Puerto Rican individuals (97.6%) were born within the 50 US states; Washington, DC; or US territories, including Puerto Rico. In the overall cohort, 7475 Hispanic/Latino individuals (58.3%) were less acculturated (SASH score less than 2). Having less than a high school level of education (4358 [32.5%] in the overall cohort) was more common among Central American (490 [39.2%]), Dominican (432 [39.8%]), Mexican (2101 [37.5%]), and Puerto Rican individuals (690 [36.4%]), while greater than a high school degree or equivalent was more common among Cuban (808 [50.8%]) and South American individuals (414 [53.2%]). When categorized into segmented groups, 5170 participants in the overall cohort (38.7%) were categorized as less acculturated and lower SES.

### Prevalence and Incidence of Diabetes and Hypertension

The overall prevalence (95% CI) of diabetes was higher at visit 2 than at visit 1 (27.6% [26.5%-28.7%] vs 17.7% [16.8%-18.6%], respectively) (Table 2).^27^ Dominican and Puerto Rican individuals had the highest prevalence (95% CI) of diabetes (19.6% [16.8%-22.3%] and 19.7% [17.2%-22.2%], respectively), while Cuban individuals had the lowest (13.8% [11.9%-15.6%]). Conversely, Mexican and Puerto Rican individuals had the highest prevalence (95% CI) of diabetes at visit 2 (31.0% [29.0%-33.0%] and 31.5% [28.6%-34.5%], respectively), whereas Cuban individuals had the lowest (21.3% [19.3%-23.4%]). Incident diabetes was highest among Mexican and Puerto Rican persons (17.2% [95% CI, 15.5%-19.0%] and 16.6% [95% CI, 13.9%-19.2%], respectively), whereas Central American and Cuban individuals had the lowest (10.2% [95% CI, 8.1%-12.3%] and 11.4% [95% CI, 10.0%-12.9%], respectively).

**Table 2.** Weighted Age-Standardized Prevalence and Incidence of Diabetes and Hypertension Overall and by National Heritage Group at Visit 1 and Visit 2: Hispanic Community Health Study/Study of Latinos^a^

Variable		Rate (95% CI), %^b^					
		Total (N = 11 623)^c^	Heritage				
			Central American (n = 1207)	Cuban (n = 1645)	Dominican (n = 1021)	Mexican (n = 4806)	Puerto Rican (n = 1801)
**Diabetes** ^d^							
Prevalence^e^	Visit 1	17.7 (16.8-18.6)	18.7 (16.1-21.2)	13.8 (11.9-15.6)	19.6 (16.8-22.3)	18.9 (17.1-20.7)	19.7 (17.2-22.2)
	Visit 2	27.6 (26.5-28.7)	24.0 (21.2-26.8)	21.3 (19.3-23.4)	28.2 (25.0-31.4)	31.0 (29.0-33.0)	31.5 (28.6-34.5)
Incidence^f^	Visit 2	14.6 (13.6-15.6)	10.2 (8.1-12.3)	11.4 (10.0-12.9)	13.1 (10.4-15.8)	17.2 (15.5-19.0)	16.6 (13.9-19.2)
**Hypertension** ^d^							
Prevalence^e^	Visit 1	27.4 (26.4-28.5)	26.2 (23.5-29.0)	30.8 (28.5-33.1)	32.7 (29.7-35.6)	22.1 (20.1-24.1)	33.0 (30.3-35.8)
	Visit 2	35.3 (34.2-36.4)	33.3 (31.0-35.7)	37.9 (36.0-39.8)	41.7 (38.8-44.6)	29.7 (27.9-31.5)	41.7 (38.4-45.0)
Incidence^f^	Visit 2	20.4 (19.0-21.9)	19.2 (15.7-22.8)	22.7 (20.1-25.4)	27.1 (22.7-31.4)	16.9 (14.8-18.9)	23.2 (19.2-27.2)

^a^ Analyses were weighted to account for complex sampling and study design of the Hispanic Community Health Study/Study of Latinos and age-adjusted to the 2010 US Census population and restricted to participants that completed visit 1 and visit 2. Visit 1 spanned 2007 to 2011, and visit 2 spanned 2014 to 2017.

^b^ Percentages are based on nonmissing values.

^c^ The 795 individuals of South American heritage and the 348 individuals who did not identify with a specific heritage or self-reported other heritage were not included in calculating the prevalence and incidence of diabetes and hypertension. These values were not calculated for the South American group due to the heterogeneous backgrounds from up to 13 different countries.

^d^ *P* values for heritage comparisons were all significant (*P* < .001).

^e^ Prevalence refers to the confirmed diagnoses of diabetes or hypertension at visit 1 and visit 2.

^f^ Incidence refers to new diagnoses of diabetes or hypertension at visit 2 in individuals without this diagnosis at visit 1.

The prevalence (95% CI) of hypertension was lower at visit 1 than at visit 2 across all heritages (27.4% [26.4%-28.5%] vs 35.3% [34.2%-36.4%], respectively).^26^ At visit 1, the prevalence (95% CI) of hypertension was highest among Puerto Rican, Dominican, and Cuban individuals (33.0% [30.3%-35.8%]; 32.7% [29.7%-35.6%]; and 30.8% [28.5%-33.1%], respectively), whereas Mexican individuals had the lowest (22.1% [20.1%-24.1%]). At visit 2, Dominican and Puerto Rican individuals continued to have the highest prevalence (95% CI) of hypertension (41.7% [38.8%-44.6%] and 41.7% [38.4%-45.0%], respectively), while Mexican individuals continued to have the lowest (29.7% [27.9%-31.5%]). Incident hypertension was highest among Dominican individuals and lowest among Mexican individuals (27.1% [95% CI, 22.7%-31.4%] vs 16.9% [95% CI, 14.8%-18.9%], respectively).

### Diabetes and Hypertension by Segmented Groups

The prevalence of diabetes was higher at visit 2 than at visit 1 across all 4 segmented groups (Table 3). At visit 1, less acculturated individuals with lower SES had the highest prevalence (95% CI) of diabetes (20.0% [18.7%-21.3%]), whereas more acculturated individuals with higher SES had the lowest (14.1% [11.8%-16.4%]). At visit 2, less acculturated individuals with lower SES continued to have the highest prevalence (95% CI) of diabetes (30.5% [28.7%-32.2%]). However, less acculturated individuals with higher SES had the lowest (22.7% [20.3%-25.0%]). Incident diabetes was highest among more acculturated individuals with lower SES and in less acculturated individuals with lower SES (16.8% [95% CI, 14.0%-19.7%] and 16.0% [95% CI, 14.3%-17.7%], respectively). The incidence was lower among more acculturated individuals with higher SES and less acculturated individuals with higher SES (12.2% [95% CI, 9.9%-14.4%] and 12.4% [95% CI, 10.2%-14.6%], respectively).

**Table 3.** Weighted, Age-Standardized Prevalence and Incidence of Diabetes and Hypertension in Segmented Groups: Hispanic Community Health Study/Study of Latinos^a^

Variable		Rate (95% CI), %^b^						
		Total (N = 11 623)^c^	Segmented group^d^					
			Less acculturated, lower SES (n = 5170)		Less acculturation, higher SES (n = 2286)		More acculturated, lower SES (n = 2067)	More acculturated, higher SES (n = 2024)
**Diabetes^e^**								
Prevalence	Visit 1	17.7 (16.8-18.6)	20.0 (18.7-21.3)	14.2 (12.3-16.1)		17.8 (15.7-20.0)		14.1 (11.8-16.4)
	Visit 2	27.6 (26.5-28.7)	30.5 (28.7-32.2)	22.7 (20.3-25.0)		29.6 (26.8-32.5)		23.2 (20.7-25.7)
Incidence	Visit 2	14.6 (13.6-15.6)	16.0 (14.3-17.7)	12.4 (10.2-14.6)		16.8 (14.0-19.7)		12.2 (9.9-14.4)
**Hypertension^e^**								
Prevalence	Visit 1	27.4 (26.4-28.5)	27.5 (26.1-28.9)	26.4 (24.4-28.4)		29.2 (26.5-31.9)		25.7 (23.1-28.3)
	Visit 2	35.3 (34.2-36.4)	36.2 (34.7-37.6)	34.2 (32.2-36.2)		38.5 (35.8-41.1)		30.0 (27.2-32.7)
Incidence	Visit 2	20.4 (19.0-21.9)	22.2 (20.4-24.1)	19.9 (17.0-22.7)		22.1 (18.4-25.7)		12.6 (10.2-15.0)

Abbreviation: SES, socioeconomic status.

^a^ Analyses were weighted to account for complex sampling and study design of the Hispanic Community Health Study/Study of Latinos and age-adjusted to the 2010 US Census population and restricted to participants that completed visit 1 and visit 2. Visit 1 spanned 2007 to 2011, and visit 2 spanned 2014 to 2017.

^b^ Percentages are based on nonmissing values.

^c^ There were 76 individuals with missing data for educational attainment only (n = 25), acculturation score only (n = 33), or both (n = 18) and were not included in calculating the prevalence and incidence of diabetes.

^d^ Acculturation was determined as low vs high based Short Acculturation Scale for Hispanics language subscale from 0 to 5, with values from 0 to less than 2 categorized as low acculturation and values from 2 to 5 categorized as high acculturation. SES was determined as low vs high using educational attainment as a proxy measure of socioeconomic status, with high school level or less categorized as low SES, and some college or more as high SES.

^e^ *P* values for segmented group comparisons were all significant (*P* < .001).

The prevalence of hypertension was also higher at visit 2 compared to visit 1 across all segmented groups. At visit 1, the prevalence of hypertension was similar across all segmented groups (range, 25.7%-29.2%). At visit 2, the prevalence (95% CI) of hypertension was highest among more acculturated individuals with lower SES and lowest among more acculturated individuals with higher SES (38.5% [35.8%-41.1%] vs 30.0% [27.2%-32.7%], respectively). Incident hypertension was highest among less acculturated individuals with lower SES and lowest in more acculturated individuals with higher SES (22.2% [95% CI, 20.4-24.1%] vs 12.6% [95% CI, 10.2%-15.0%], respectively).

### Adjusted Models of Incident Diabetes and Hypertension

Overall, more acculturated participants with higher SES had a significantly lower predicted probability of diabetes (WPP, 0.11 [95% CI, 0.09-0.13]) compared to less acculturated individuals with lower SES (WPP, 0.17 [95% CI, 0.14-0.19]) (Table 4). Similarly, more acculturated participants with higher SES had a significantly lower predicted probability of hypertension (WPP, 0.10 [95% CI, 0.08-0.12]) compared to both less acculturated individuals with lower SES (WPP, 0.18 [95% CI, 0.15-0.21]) and more acculturated individuals with lower SES (WPP, 0.19 [95% CI, 0.15-0.23]). Comparison of predicted probabilities by heritage group lacked statistical significance. In general, less acculturated individuals with lower SES groups had a higher predicted probability of incident diabetes and hypertension, with the exception of Central American individuals.

**Table 4.** Weighted Predicted Probabilities from Modeling Incident Diabetes and Incident Hypertension for Segmented Groups Stratified by HeritageHispanic Community Health Study/Study of Latinos^a^

Variable	Weighted predicted probability (95% CI)							
	Total (N = 11 623)	Heritage^b^						
		Central American (n = 1207)		Cuban (n = 1645)		Dominican (n = 1021)	Mexican (n = 4806)	Puerto Rican (n = 1801)
**Incident diabetes by segmented group** ^c^ ^,^ ^d^								
Less acculturated, lower SES	0.17 (0.14-0.19)	0.09 (0.04-0.18)	0.17 (0.10-0.26)		0.15 (0.07-0.30)		0.15 (0.09-0.22)	0.17 (0.07-0.36)
Less acculturated, higher SES	0.14 (0.12-0.17)	0.11 (0.04-0.24)	0.16 (0.09-0.26)		0.12 (0.04-0.31)		0.11 (0.07-0.18)	0.07 (0.01-0.29)
More acculturated, lower SES	0.14 (0.11-0.16)	0.04 (0.01-0.12)	0.11 (0.06-0.20)		0.05 (0.01-0.17)		0.15 (0.10-0.23)	0.12 (0.04-0.27)
More acculturated, higher SES	0.11 (0.09-0.13)	0.10 (0.04-0.25)	0.09 (0.05-0.15)		0.07 (0.03-0.16)		0.10 (0.06-0.17)	0.09 (0.03-0.22)
**Incident hypertension by segmented group** ^b^ ^,^ ^c^								
Less acculturated, lower SES	0.18 (0.15-0.21)	0.12 (0.08-0.16)	0.24 (0.12-0.42)		0.20 (0.10-0.37)		0.13 (0.08-0.20)	0.26 (0.14-0.42)
Less acculturated, higher SES	0.15 (0.12-0.18)	0.07 (0.04-0.13)	0.23 (0.12-0.40)		0.12 (0.06-0.24)		0.09 (0.05-0.15)	0.22 (0.09-0.45)
More acculturated, lower SES	0.19 (0.15-0.23)	0.14 (0.06-0.28)	0.17 (0.07-0.34)		0.15 (0.07-0.29)		0.15 (0.09-0.24)	0.21 (0.11-0.36)
More acculturated, higher SES	0.10 (0.08-0.12)	0.11 (0.06-0.20)	0.11 (0.05-0.24)		0.13 (0.06-0.27)		0.08 (0.04-0.14)	0.10 (0.05-0.20)

Abbreviation: SES, socioeconomic status.

^a^ Analyses were weighted to account for complex sampling and study design of the Hispanic Community Health Study/Study of Latinos and age-adjusted to the 2010 US Census population and restricted to participants that completed visit 1 and visit 2. Visit 1 spanned 2007 to 2011, and visit 2 spanned 2014 to 2017. Those who were missing heritage groups or segments in the model as well as those missing model covariates were excluded from this analysis.

^b^ Predicted probabilities were not calculated for the South American group due to the heterogeneous backgrounds from up to 13 different countries.

^c^ Acculturation was dichotomized as low vs high based on the Short Acculturation Scale for Hispanics language subscale from 0 to 5, with values from 0 to 1.9 categorized as low acculturation and values from 2 to 5 categorized as high acculturation. SES was dichotomized as low vs high using educational attainment as a proxy measure of SES, with a high school level of education or less categorized as low SES, and some college or more categorized as high SES.

^d^ Regression models adjusted for age, gender, health insurance status, study site, family history of diabetes and hypertension, obesity, poor diet, physical activity, and birthplace (except for models for Cuban and Dominican heritages, which did not adjust for study site for the incident diabetes analysis, and Central American heritage, which did not adjust for birthplace for the incident hypertension analysis).

### Sensitivity Analysis

Additional measures of acculturation and socioeconomic factors assessed at visit 1 were considered in the sensitivity analysis and are shown in eTable 1 in Supplement 1. Selected sensitivity analyses were reported in eTables 2 and 3 in Supplement 1. Whether years in the US (<10 years vs ≥10 years) would modify, via stratification, the modeling results were also assessed; however, results generally did not differ from nonstratified results (eTable 4 in Supplement 1). The results of the sensitivity analysis incorporating visit 2 data did not differ significantly from the main results (eTables 5 and 6 in Supplement 1). Overall, the sensitivity analyses yielded similar results as the reported main outcomes using selected acculturation and SES variables.

## Discussion

Prior studies have suggested that increases in acculturation may have a deleterious effect on cardiometabolic health and contribute to the loss of potential health advantages from which Hispanic/Latino immigrant groups initially benefit compared to their US-born counterparts.^8,10,35^ However, we found that participants in the HCHS/SOL cohort who were from higher SES had a lower prevalence of diabetes and hypertension at visit 2, despite acculturation level and after adjusting for lifestyle factors such as diet quality, physical activity, and obesity at visit 1. Furthermore, participants with higher SES also had a lower predicted probability of incident diabetes and hypertension at visit 2. This observation suggests that having educational attainment beyond high school may be protective of incident cardiometabolic disease among individuals from diverse Hispanic/Latino heritages. This finding is consistent with previous literature, which has observed that Hispanic/Latino individuals with an education greater than high school have a lower prevalence of diabetes,^36^ and within the HCHS/SOL sample, overall cardiovascular risk factors and hypertension improved with higher SES.^2,26,29^ It is possible that this combination of more acculturated persons with higher SES may reflect greater ease with both daily life in US society because of language fluency and sense of socioeconomic security, which may lessen chronic stress. It appears evident from these data that social mobility, as defined by years of education, has an important relationship with health outcomes that needs to be considered in analyses of the associations with acculturation. In fact, participants with lower SES had a higher probability of both diabetes and hypertension than their counterparts with more than high school education in either acculturation category.

Few previous studies have investigated whether SES moderates the relationship between acculturation level and incident diabetes and hypertension in Hispanic/Latino populations. Using the segmented assimilation model, we categorized participants into groups based on their level of acculturation and SES. This decision was not only supported by empirical evidence,^17,18^ but also rooted in the theory that segmented assimilation offers the opportunity to identify what factors prevent some immigrant groups, and not others, from becoming more susceptible to adverse outcomes.^31^ Our findings provide new insights into the relationship between acculturation, SES, and diabetes/hypertension risk among Hispanic/Latino individuals by national background.

Studies that have explored the association of immigration and acculturation factors with various cardiometabolic health outcomes in Hispanic/Latino populations have shown mixed results, especially regarding the relationship between acculturation and diabetes.^15,16,19,24^ Building on previous research that evaluates Hispanic/Latino individuals as an aggregate group or focuses most often on Mexican individuals,^12,13,17,18,21^ we found incident rates of diabetes and hypertension varied significantly by heritage,^19,25,26^ but overall rates were alarmingly high for all subpopulations. Overall, diabetes was present in 27.6% and hypertension in 35.3% of participants at visit 2, reflecting the magnitude of these chronic conditions among Hispanic/Latino populations. Although our findings were consistent with previous research, they build on past cross-sectional analyses and suggest that there is a need to better understand the social, behavioral, and contextual factors that account for these differences in health outcomes.

### Strengths and Limitations

A strength of this study was that we used longitudinal data and were able to calculate the incidence of diabetes and hypertension in a well-characterized probabilistic sample of a community-based cohort of Hispanic/Latino individuals. This is especially important when investigating acculturation and SES, using the segmented assimilation model at large. Definitions of diagnosed diabetes and hypertension used standard criteria by self-report, laboratory results, and medication data, unlike many other studies that rely solely on self-report.^17^

A limitation of the HCHS/SOL is that its design did not include a higher proportion of US-born Hispanic/Latino persons. The overall Hispanic/Latino population in the US is 70% US born, 62% of Mexican heritage, and on average younger than the individuals in HCHS/SOL. Diet quality and physical activity were also not measured at visit 2, and it is possible that differential changes in these factors may have confounded the results by segmented groups.

## Conclusions

Advancing our knowledge of the relationship between acculturation and SES and incident diabetes and hypertension in a diverse population of Hispanic/Latino individuals is critical to help identify factors associated with the risk of cardiometabolic disease. The findings of this cohort study suggest that SES measured by educational attainment may be a more important factor influencing cardiometabolic health than level of acculturation although these 2 measures are correlated. The impact of acculturation on cardiometabolic health may vary by segmented groups, where the preservation of a community’s values and identity may not protect from developing diabetes in the absence of improved economic opportunities. In the absence of other data, the assumption that becoming more acculturated has an adverse effect on Hispanic/Latino health outcomes should be considered an oversimplification. Health care systems, clinicians, and policymakers should recognize how the interaction between heritage, acculturation, and SES can inform the development of prevention and treatment strategies associated with cardiometabolic health among Hispanic/Latino individuals, which remains a major health crisis for these communities.

## Notes

Supplement 1. eTable 1. Additional weighted, age-standardized measures of acculturation and socioeconomic status: Hispanic Community Health Study/Study of Latinos (n=11,623), (2007-2011) eTable 2. Weighted predicted probabilities from modeling incident diabetes and incident hypertension for segmented groups using selected measures of perceived SES and income stratified by heritage as a sensitivity analysis: Hispanic Community Health Study/Study on Latinos (n=11,623), ([2008-2011], [2014-2017]) eTable 3. Weighted predicted probabilities from modeling incident diabetes and incident hypertension for segmented groups using selected measures of years lived in the US and perceived SES stratified by heritage as a sensitivity analysis: Hispanic Community Health Study/Study on Latinos (n=11,623), ([2008-2011], [2014-2017]) eTable 4. Weighted predicted probabilities from modeling results of incident diabetes and incident hypertension in segmented groups (using main analysis variables of SASH language score and education), stratified by <10 years and ≥10 years lived in the US as a sensitivity analysis: Hispanic Community Health Study/Study on Latinos (n=11,623), ([2008-2011], [2014-2017]) eTable 5. Weighted predicted probabilities from modeling incident diabetes and incident hypertension at Visit 2 using variables at Visit 2, as a sensitivity analysis, in segmented groups stratified by heritage: Hispanic Community Health Study/Study on Latinos (n=11,623), ([2008-2011], [2014-2017]) eTable 6. Weighted predicted probabilities from modeling incident diabetes and incident hypertension at Visit 2 using change in control variables between Visit 1 and Visit 2, as a sensitivity analysis, in segmented groups stratified by heritage: Hispanic Community Health Study/Study on Latinos (n=11,623), ([2008-2011], [2014-2017])

Supplement 2. Data sharing statement
