# Supplementary material for: Diabetes and Hypertension Risk Across Acculturation and Education Levels in Hispanic/Latino Adults: The Hispanic Community Health Study/Study of Latinos
Source: JAMA Health Forum. 2025 Mar 28;6(3):e250273. doi: 10.1001/jamahealthforum.2025.0273 (PMC11953754; doi:10.1001/jamahealthforum.2025.0273)
Supplement: Supplement 1. — eTable 1. Additional weighted, age-standardized measures of acculturation and socioeconomic status: Hispanic Community Health Study/Study of Latinos (n=11,623), (2007-2011) eTable 2. Weighted predicted probabilities from modeling incident diabetes and incident hypertension for segmented groups using selected measures of perceived SES and income stratified by heritage as a sensitivity analysis: Hispanic Community Health Study/Study on Latinos (n=11,623), ([2008-2011], [2014-2017]) eTable 3. Weighted predicted probabilities from modeling incident diabetes and incident hypertension for segmented groups using selected measures of years lived in the US and perceived SES stratified by heritage as a sensitivity analysis: Hispanic Community Health Study/Study on Latinos (n=11,623), ([2008-2011], [2014-2017]) eTable 4. Weighted predicted probabilities from modeling results of incident diabetes and incident hypertension in segmented groups (using main analysis variables of SASH language score and education), stratified by <10 years and ≥10 years lived in the US as a sensitivity analysis: Hispanic Community Health Study/Study on Latinos (n=11,623), ([2008-2011], [2014-2017]) eTable 5. Weighted predicted probabilities from modeling incident diabetes and incident hypertension at Visit 2 using variables at Visit 2, as a sensitivity analysis, in segmented groups stratified by heritage: Hispanic Community Health Study/Study on Latinos (n=11,623), ([2008-2011], [2014-2017]) eTable 6. Weighted predicted probabilities from modeling incident diabetes and incident hypertension at Visit 2 using change in control variables between Visit 1 and Visit 2, as a sensitivity analysis, in segmented groups stratified by heritage: Hispanic Community Health Study/Study on Latinos (n=11,623), ([2008-2011], [2014-2017]) [file jamahealthforum-e250273-s001.pdf]

Supplemental Online Content

Pérez-Stable EJ, Panigrahi A, Coreas SI, et al. Diabetes and hypertension risk across acculturation and education levels in Hispanic/Latino adults. *JAMA Health Forum.* 2025;6(3):e250273. doi:10.1001/jamahealthforum.2025.0273

eTable 1. Additional weighted, age-standardized measures of acculturation and socioeconomic status: Hispanic Community Health Study/Study of Latinos (n=11,623), (2007-2011)

eTable 2. Weighted predicted probabilities from modeling incident diabetes and incident hypertension for segmented groups using selected measures of perceived SES and income stratified by heritage as a sensitivity analysis: Hispanic Community Health Study/Study on Latinos (n=11,623), ([2008-2011], [2014-2017])

eTable 3. Weighted predicted probabilities from modeling incident diabetes and incident hypertension for segmented groups using selected measures of years lived in the US and perceived SES stratified by heritage as a sensitivity analysis: Hispanic Community Health Study/Study on Latinos (n=11,623), ([2008-2011], [2014-2017])

eTable 4. Weighted predicted probabilities from modeling results of incident diabetes and incident hypertension in segmented groups (using main analysis variables of SASH language score and education), stratified by <10 years and ≥10 years lived in the US as a sensitivity analysis: Hispanic Community Health Study/Study on Latinos (n=11,623), ([2008-2011], [2014-2017])

eTable 5. Weighted predicted probabilities from modeling incident diabetes and incident hypertension at Visit 2 using variables at Visit 2, as a sensitivity analysis, in segmented groups stratified by heritage: Hispanic Community Health Study/Study on Latinos (n=11,623), ([2008-2011], [2014-2017])

eTable 6. Weighted predicted probabilities from modeling incident diabetes and incident hypertension at Visit 2 using change in control variables between Visit 1 and Visit 2, as a sensitivity analysis, in segmented groups stratified by heritage: Hispanic Community Health Study/Study on Latinos (n=11,623), ([2008-2011], [2014-2017])

This supplemental material has been provided by the authors to give readers additional information about their work.

© 2025 Pérez-Stable EJ et al. *JAMA Health Forum*.

eTable 1. Additional weighted, age-standardized measures of acculturation and socioeconomic status that are not shown in main Table 1, but used for sensitivity analyses, stratified by heritage at Visit 1: Hispanic Community Health Study/Study of Latinos (n=11,623), (2007-2011)*

Characteristics	Total** *n*=11,623		Heritage** Central American *n*=1,207		Cuban *n*=1,645		Dominican *n*=1,021		Mexican *n*=4,806		Puerto Rican *n*=1,801		South American *n*=795	
	%[a]	95% CI	%[a]	95% CI	%[a]	95% CI	%[a]	95% CI	%[a]	95% CI	%[a]	95% CI	%[a]	95% CI
**Age**, mean (SE)	43.1	(42.5,43.7)	41.7	(40.6,42.9)	49.2	(48.1,50.3)	41.9	(40.6,43.3)	40.1	(39.3,40.9)	45.2	(44.0,46.4)	44.2	(42.5,45.9)
**Years in US**														
Less than 6 years	16.9	(15.3,18.6)	19.6	(16.4,22.7)	38.2	(34.1,42.4)	16.8	(13.4,20.1)	11.2	(9.3,13.2)	4.7	(2.9,6.6)	16.3	(12.1,20.5)
6 years or more	83.4	(81.8,85.1)	80.7	(77.7,83.8)	61.9	(57.7,66.0)	84.0	(80.7,87.4)	88.8	(86.9,90.8)	95.3	(93.4,97.1)	83.7	(79.5,87.9)
Less than 10 years	27.4	(25.4,29.5)	31.9	(27.9,35.9)	52.4	(47.8,57.1)	26.1	(21.9,30.2)	22.0	(19.6,24.4)	6.9	(4.7,9.2)	37.6	(31.8,43.4)
10 years or more	72.6	(70.5,74.6)	68.1	(64.1,72.1)	47.6	(43.0,52.2)	73.9	(69.8,78.1)	78.0	(75.6,80.4)	93.1	(90.8,95.4)	62.4	(56.6,68.2)
Years lived in the U.S., mean (SE)	20.7	(19.1,21.4)	16.4	(15.6,17.3)	12.9	(11.6,14.2)	17.8	(16.8,18.8)	21.9	(21.2,22.7)	33.7	(32.7,34.7)	15.5	(14.3,16.6)
**Immigrant Generation[b]**														
First generation	78.5	(76.9,80.0)	93.8	(91.8,95.8)	90.6	(87.6,93.5)	90.2	(87.5,92.9)	76.6	(74.3,78.9)	51.4	(48.1,54.8)	93.9	(91.1,96.8)
Second generation and beyond	21.5	(20.0,23.1)	6.2	(4.2,8.2)	9.4	(6.5,12.4)	9.8	(7.1,12.5)	23.4	(21.1,25.7)	48.6	(45.2,51.9)	6.1	(3.2,8.9)
**Language Preference**														
Spanish	77.5	(75.7,79.2)	89.0	(86.1,91.9)	90.5	(87.7,93.3)	82.8	(79.5,86.2)	81.1	(79.0,83.3)	41.6	(37.2,46.0)	89.1	(85.8,92.5)
English	22.5	(20.8,24.3)	11.0	(8.1,13.9)	9.5	(6.7,12.3)	17.2	(13.8,20.6)	18.9	(16.7,21.0)	58.4	(54.0,62.8)	10.9	(7.6,14.3)
**Acculturation SASH Scale[b] (1-5)**														
Low <2	58.3	(56.4,60.3)	72.9	(68.6,77.1)	74.8	(70.9,78.7)	65.0	(60.8,69.1)	59.9	(57.6,62.3)	22.2	(19.1,25.2)	63.3	(59.3,67.4)
Moderate 2-2.9	18.1	(17.0,19.3)	16.0	(13.0,19.1)	13.1	(10.5,15.8)	20.2	(16.7,23.8)	20.3	(18.3,22.2)	17.9	(15.3,20.6)	24.8	(20.7,29.0)
High ≥3	23.5	(21.8,25.2)	11.1	(8.1,14.1)	12.0	(8.5,15.5)	14.8	(11.7,17.9)	19.8	(17.9,21.8)	59.9	(56.4,63.4)	11.8	(8.8,14.9)
**Ethnic identification**														
Low (1.0-2.9)	15.5	(14.4,16.6)	10.2	(8.0,12.3)	9.2	(6.9,11.4)	13.8	(11.0,16.5)	17.6	(15.8,19.3)	21.9	(18.7,25.1)	15.6	(12.4,18.8)
Moderate (3.0)	47.0	(45.7,48.4)	47.7	(43.8,51.6)	50.6	(47.1,54.1)	43.1	(39.2,47.2)	47.8	(45.7,50.0)	45.6	(42.0,49.2)	41.7	(37.0,46.3)
High (3.1-4.0)	37.5	(36.1,38.8)	42.1	(38.3,45.9)	40.2	(36.6,43.9)	43.1	(39.3,46.9)	34.6	(32.7,36.6)	32.5	(29.4,35.6)	42.8	(38.0,47.6)
**Education (4-level)**														
Less than high school	32.5	(30.9,34.1)	39.2	(35.3,43.1)	20.5	(17.9,23.1)	39.8	(35.8,43.9)	37.5	(34.6,40.4)	36.4	(32.3,40.6)	20.4	(16.5,24.3)
High school graduate or equivalent	26.8	(25.6,28.0)	22.8	(19.9,25.7)	28.7	(25.4,32.1)	21.5	(18.3,24.8)	27.7	(25.9,29.5)	26.8	(23.8,29.8)	26.4	(22.5,30.4)
College (trade, vocational, associate, bachelor)	37.3	(35.7,39.0)	34.6	(30.6,38.6)	46.8	(43.0,50.6)	35.8	(31.8,39.8)	31.8	(29.1,34.5)	33.7	(30.1,37.4)	48.6	(43.8,53.4)
Post-College (master, professional, doctorate)	3.4	(2.6,4.2)	3.4	(1.7,5.0)	4.0	(2.7,5.3)	2.8	(1.5,4.1)	3.0	(1.6,4.4)	3.0	(1.6,4.5)	4.6	(2.7,6.5)

© 2025 Pérez-Stable EJ et al. *JAMA Health forum*.

**Household Income**														
< $10,001	15.1	(13.8,16.4)	14.9	(11.5,18.2)	18.3	(15.4,21.2)	18.1	(14.9,21.3)	10.7	(9.0,12.5)	21.5	(17.6,25.5)	10.2	(7.5,12.9)
$10,001 - $20,000	31.7	(30.1,33.4)	36.3	(31.7,40.8)	34.1	(30.1,38.1)	36.1	(31.6,40.6)	28.6	(25.8,31.4)	30.5	(26.8,34.2)	36.7	(31.7,41.6)
$20,001 - $40,000	33.1	(31.6,34.7	35.6	(31.2,40.1)	32.4	(28.7,36.2)	33.4	(29.3,37.6)	36.3	(33.8,38.8)	26.4	(22.8,30.0)	32.1	(27.4,36.9)
$40,001 - $75,000	14.6	(13.2,16.1)	10.9	(8.1,13.7)	10.0	(7.6,12.5)	10.5	(7.2,13.7)	17.6	(15.2,20.0)	14.9	(12.3,17.6)	14.5	(10.5,18.5)
> $75,000	5.4	(4.3,6.5)	2.3	(1.2,3.4)	5.1	(2.4,7.9)	1.9	(0.8,3.0)	6.7	(5.0,8.5)	6.6	(4.8,8.5)	6.5	(3.4,9.6)
**Health Insurance Type**														
No health insurance	48.1	(46.2,50.1)	65.5	(60.9,70.1)	59.3	(55.8,62.8)	25.8	(21.8,29.8)	54.5	(51.8,57.2)	18.8	(15.8,21.9)	57.7	(52.9,62.5)
Public insurance only	21.6	(20.2,23.0)	12.8	(10.5,15.0)	23.8	(20.9,26.8)	35.2	(31.1,39.4)	12.5	(10.6,14.3)	40.4	(36.2,44.6)	12.8	(9.5,16.1)
Any private insurance	24.5	(22.6,26.5)	16.3	(12.8,19.7)	12.7	(9.8,15.6)	35.2	(30.9,39.5)	25.0	(22.3,27.6)	37.1	(33.3,40.9)	23.6	(19.3,27.8)
Unknown insurance type	5.8	(5.0,6.6)	5.5	(3.9,7.1)	4.2	(2.7,5.6)	3.8	(1.9,5.6)	8.1	(6.6,9.5)	3.7	(2.5,4.9)	5.9	(2.8,9.0)
**Employment**														
Retired and unemployed	10.2	(9.5,10.9)	7.2	(5.4,9.1)	8.3	(7.2,9.4)	12.3	(10.3,14.4)	8.3	(7.2,9.4)	17.1	(15.4,18.9)	7.4	(5.3,9.5)
Not retired and unemployed	39.8	(38.3,41.4)	34.9	(31.3,38.4)	47.2	(43.4,51)	39.5	(34.5,44.6)	36.7	(34.5,39)	44.1	(40.4,47.7)	31.9	(26.9,36.9)
Employed part-time (≤ 35 hrs/wk)	16.8	(15.9,17.8)	22.8	(19.7,25.9)	13.3	(11.2,15.4)	17.2	(13.8,20.5)	19.3	(17.7,20.9)	10.0	(7.8,12.3)	26.5	(22.2,30.8)
Employed full-time (> 35 hrs/wk)	33.2	(31.9,34.5)	35.1	(31.5,38.7)	31.3	(28.1,34.4)	31.0	(26.7,35.2)	35.7	(33.5,37.9)	28.8	(25.4,32.2)	34.2	(30.3,38.2)
**Current Occupation**														
Non-skilled worker	27.0	(25.1,28.9)	27.1	(22.9,31.3)	13.6	(10.5,16.6)	28.7	(22.4,34.9)	35.1	(31.6,38.5)	22.4	(17.5,27.4)	20.7	(15.1,26.3)
Service worker	20.5	(18.3,22.6)	32.4	(27.9,36.8)	35.8	(30.3,41.3)	18.4	(14.5,22.3)	14.0	(11.1,16.8)	9.1	(6.5,11.7)	30.1	(23.7,36.4)
Skilled worker	21.6	(19.7,23.5)	13.0	(9.3,16.8)	15.5	(11.8,19.1)	26.5	(21.2,31.8)	24.5	(21.4,27.6)	26.2	(21.4,31.1)	17.2	(13.1,21.3)
Professional/Admin/Office staff	13.7	(11.8,15.6)	13.0	(9.4,16.5)	12.5	(8.4,16.7)	7.7	(4.9,10.4)	13.3	(10.3,16.2)	21.0	(16.2,25.9)	14.1	(10,18.1)
Other occupation	17.2	(15.5,18.9)	14.6	(10.9,18.2)	22.7	(18.7,26.7)	18.8	(14.4,23.2)	13.2	(11.2,15.3)	21.2	(15.8,26.7)	18.1	(13.4,22.7)
**Assets – House / Apartment / Mobile home**														
Owned, without mortgage or loan	6.7	(5.8,7.6)	3.6	(2.2,5.1)	4.7	(3.3,6.1)	2.4	(0.8,3.9)	10.1	(8.4,11.8)	5.6	(3.5,7.7)	5.3	(3.0,7.6)
Owned, with mortgage or loan	20.9	(18.7,23)	13.1	(10,16.2)	14.9	(11.7,18.1)	7.9	(4.2,11.6)	31.3	(28.1,34.4)	16.1	(13.0,19.2)	24.3	(19.1,29.5)
Rented	70.0	(67.5,72.4)	80.5	(77,84.1)	78.5	(74.9,82.1)	86.3	(81.7,91)	56.7	(53.1,60.3)	75.4	(71.6,79.1)	66.7	(61.3,72.2)
Occupied without payment	2.5	(2.1,2.9)	2.7	(1.5,4.0)	1.9	(1.3,2.5)	3.4	(2.0,4.9)	2.0	(1.4,2.6)	2.9	(1.8,4.1)	3.7	(2.0,5.4)
**Perceived SES Ladder[c] (1-10)**														
Low (1 - 3)	30.5	(29,32.1)	31.1	(27.4,34.8)	34.2	(31.1,37.4)	45.8	(41.4,50.2)	26.7	(24.0,29.4)	31.1	(27.7,34.6)	22.3	(18.3,26.4)
Average (4 - 6)	57.7	(56.4,59.1)	59.5	(55.5,63.6)	58.7	(55.8,61.7)	47.0	(42.9,51.1)	59.5	(57.2,61.9)	54.4	(50.5,58.2)	62.9	(58.2,67.6)
High (7 - 10)	11.8	(10.7,12.9)	9.4	(6.8,12.0)	7.0	(4.9,9.2)	7.2	(5.0,9.4)	13.8	(12.1,15.6)	14.5	(11.6,17.5)	14.8	(10.7,18.8)

© 2025 Pérez-Stable EJ et al. *JAMA Health forum*.

Perceived SES Ladder, mean (SE)	4.4	(4.3,4.5)	4.3	(4.1,4.4)	4.1	(4.0,4.3)	3.8	(3.7,4.0)	4.6	(4.5,4.7)	4.4	(4.3,4.6)	4.7	(4.5,4.9)
**Segmented Groups[d]**														
Low Acculturation, Low SES	23.2	(21.9,24.5)	34.0	(30.1,38.0)	16.1	(13.9,18.3)	32.7	(28.8,36.6)	28.9	(26.4,31.4)	11.5	(9.4,13.6)	18.8	(15.1,22.5)
Low Acculturation, High SES	35.2	(33.3,37.1)	38.8	(35.3,42.3)	58.7	(54.9,62.6)	32.3	(27.8,36.7)	31.0	(28.5,33.6)	10.7	(8.6,12.7)	44.5	(40.3,48.8)
High Acculturation., Low SES	9.3	(8.3,10.3)	5.1	(3.5,6.8)	4.4	(2.8,6.0)	7.1	(5.0,9.3)	8.6	(7.3,10.0)	24.7	(20.6,28.8)	1.8	(0.7,2.9)
High Acculturation, High SES	32.4	(30.6,34.1)	22.0	(18.1,26.0)	20.8	(17,24.6)	27.9	(23.9,31.9)	31.5	(29.2,33.8)	53.1	(48.7,57.6)	34.9	(30.7,39.1)

Note: SASH = Short Acculturation Scale for Hispanics, SE = standard error, SES = socioeconomic status

*Analyses were weighted to account for complex sampling and study design of HCHS/SOL, age-standardized to the 2010 US Census population, and restricted to participants that completed Visit 1 and Visit 2 of HCHS/SOL.

***p* values for all groups <.0001

[a]Percentages based on non-missing values.

[b]Participants were asked to respond on a 5-point Likert-type scale from 1=Only Spanish and 5=Only English to the following: (1) "*In general, what language(s) do you read and speak?"* (2) *"What was the language(s) you used as a child?"* (3) *"What language(s) do you usually speak at home?"* (4) *"In which language(s) do you usually think?"* (5) *"What language(s) do you usually speak with your friends?"* (6) *"In general, what language(s) are the movies, T.V. and radio programs you prefer to watch and listen to?"* The average of the 6 questions indicates the degree of language-based acculturation, with a higher value reflecting greater acculturation.

[c]Participants were shown a 10-step ladder and asked to respond to the following: "*Where would you place yourself on this ladder? Please show the step where you think you stand at this time in your life, relative to other people in the United States?"*

[d]Low versus high acculturation was determined by a two-level Acculturation SASH Scale variable. Low versus high SES was determined by using educational attainment as a proxy measure, with less than high school level as low SES and greater than or equal to high school level as high SES.

© 2025 Pérez-Stable EJ et al. *JAMA Health forum*.

eTable 2. Weighted predicted probabilities from modeling incident diabetes and incident hypertension for segmented groups using selected measures of perceived SES and income stratified by heritage as a sensitivity analysis: Hispanic Community Health Study/Study on Latinos (n=11,623), ([2008-2011], [2014-2017])1

		Heritage				
	**Total** ***n*=11,623**	**Central American** ***n*=1,207**	**Cuban** ***n*=1,645**	**Dominican** ***n*=1,021**	**Mexican** ***n*=4,806**	**Puerto Rican** ***n*=1,801**
**Segmented Groups[2]**	**Incident Diabetes (95% CI)[3]**					
Low Perceived SES, Low Income	0.15(0.13,0.17)	0.08(0.03,0.19)	0.11(0.07,0.18)	0.13(0.05,0.30)	0.13(0.08,0.21)	0.14(0.06,0.32)
Low Perceived SES, High Income	0.11(0.08,0.15)	0.06(0.02,0.19)	0.07(0.03,0.18)	0.04(0.01,0.15)	0.11(0.05,0.21)	0.12(0.04,0.31)
High Perceived SES, Low Income	0.14(0.12,0.16)	0.09(0.04,0.19)	0.17(0.11,0.25)	0.08(0.04,0.17)	0.11(0.06,0.17)	0.10(0.04,0.24)
High Perceived SES, High Income	0.13(0.11,0.15)	0.08(0.03,0.17)	0.10(0.06,0.15)	0.04(0.01,0.12)	0.11(0.06,0.19)	0.14(0.06,0.30)
**Segmented Groups[2]**	**Incident Hypertension (95% CI)[3]**					
Low Perceived SES, Low Income	0.17(0.14,0.21)	0.16(0.10,0.23)	0.21(0.12,0.34)	0.14(0.08,0.24)	0.12(0.08,0.16)	0.28(0.15,0.46)
Low Perceived SES, High Income	0.16(0.11,0.22)	0.09(0.02,0.32)	0.25(0.11,0.48)	0.09(0.03,0.27)	0.11(0.07,0.18)	0.19(0.07,0.43)
High Perceived SES, Low Income	0.16(0.14,0.19)	0.10(0.06,0.16)	0.17(0.08,0.32)	0.20(0.11,0.34)	0.14(0.11,0.18)	0.17(0.08,0.31)
High Perceived SES, High Income	0.13(0.11,0.15)	0.10(0.05,0.18)	0.18(0.09,0.32)	0.17(0.09,0.32)	0.08(0.06,0.11)	0.17(0.08,0.31)

Note. CI = confidence interval, SES = socioeconomic status

[1]Analyses were weighted to account for complex sampling and study design of HCHS/SOL, age-standardized to the 2010 US Census population, and restricted to participants that completed Visit 1 and Visit 2 of HCHS/SOL.

[2]Perceived SES was dichotomized as low versus high based on the perceived SES scale of 1-10, with values of 1-3 categorized as low perceived SES and values of 4-10 categorized as high perceived SES. Income was dichotomized as low versus high based on self-reported income with <$30,000 categorized as low income and ≥$30,000 categorized as high income.

[3]Regression models were adjusted for age, gender, health insurance status, study site, family history of diabetes/hypertension respectively, obesity, poor diet, physical activity, and birthplace (with the exception of models for (a) Cuban and Dominican heritage, which did not adjust for study site for incident diabetes, (b) Mexican heritage, which did not adjust for study site, and (b) Central American heritage, which did not adjust for birthplace for incident hypertension).

© 2025 Pérez-Stable EJ et al. *JAMA Health forum*.

eTable 3. Weighted predicted probabilities from modeling incident diabetes and incident hypertension for segmented groups using selected measures of years lived in the US and perceived SES stratified by heritage as a sensitivity analysis: Hispanic Community Health Study/Study on Latinos (n=11,623), ([2008-2011], [2014-2017])1

		Heritage				
	**Total** *n*=11,623	**Central American** *n*=1,207	**Cuban** *n*=1,645	**Dominican** *n*=1,021	**Mexican** *n*=4,806	**Puerto Rican** *n*=1,801
**Segmented Groups[2]**	**Incident Diabetes (95% CI)[3]**					
<10 Years in US, Low Perceived SES	0.14(0.11,0.17)	0.12(0.07,0.20)	0.11(0.07,0.17)	0.12(0.04,0.27)	0.12(0.07,0.19)	0.18(0.05,0.46)
<10 Years in US, High Perceived SES	0.14(0.08,0.23)	0.08(0.02,0.28)	0.17(0.07,0.35)	0.11(0.02,0.44)	0.11(0.04,0.27)	0.16(0.02,0.7)
≥10 Years in US, Low Perceived SES	0.14(0.13,0.15)	0.07(0.04,0.11)	0.13(0.09,0.19)	0.10(0.04,0.20)	0.13(0.09,0.20)	0.10(0.04,0.23)
≥10 Years in US, High Perceived SES	0.14(0.11,0.17)	0.12(0.07,0.21)	0.12(0.05,0.26)	0.04(0.02,0.11)	0.15(0.10,0.22)	0.08(0.03,0.2)
**Segmented Groups[2]**	**Incident Hypertension (95% CI)[3]**					
<10 Years in US, Low Perceived SES	0.14(0.11,0.17)	0.08(0.05,0.15)	0.18(0.09,0.32)	0.09(0.04,0.18)	0.12(0.07,0.20)	0.21(0.08,0.45)
<10 Years in US, High Perceived SES	0.13(0.08,0.20)	0.06(0.01,0.24)	0.24(0.08,0.52)	0.09(0.01,0.48)	0.08(0.03,0.17)	0.35(0.05,0.86)
≥10 Years in US, Low Perceived SES	0.17(0.15,0.19)	0.12(0.08,0.17)	0.19(0.11,0.32)	0.17(0.10,0.28)	0.13(0.08,0.20)	0.21(0.10,0.37)
≥10 Years in US, High Perceived SES	0.11(0.09,0.14)	0.12(0.06,0.22)	0.09(0.03,0.25)	0.14(0.07,0.27)	0.08(0.04,0.14)	0.12(0.05,0.27)

Note. CI = confidence interval, SES = socioeconomic status

[1]Analyses were weighted to account for complex sampling and study design of HCHS/SOL, age-standardized to the 2010 US Census population, and restricted to participants that completed Visit 1 and Visit 2 of HCHS/SOL.

[2]Years in the US was dichotomized as <10 years versus ≥10 years based on self-report and used as the measure of acculturation. Perceived SES was dichotomized as low versus high based on the perceived SES scale of 1-10, with values of ≤5 categorized as low perceived SES and values >5 categorized as high perceived SES.

[3]Regression models were adjusted for age, gender, health insurance status, study site, family history of diabetes/hypertension respectively, obesity, poor diet, physical activity, and birthplace (with the exception of models for (a) Cuban and Dominican heritage, which did not adjust for study site for incident diabetes and (b) Central American heritage, which did not adjust for birthplace for incident hypertension).

© 2025 Pérez-Stable EJ et al. *JAMA Health forum*.

eTable 4. Weighted predicted probabilities from modeling results of incident diabetes and incident hypertension in segmented groups (using main analysis variables of SASH language score and education), stratified by <10 years and ≥10 years lived in the US as a sensitivity analysis: Hispanic Community Health Study/Study on Latinos (n=11,623), ([2008-2011], [2014-2017])1

	Total<br>n = 11,623	<10 Years in the US<br>n = 2,629	10 or more years in the US<br>n = 8,941
**Segmented Groups[2]**	**Incident Diabetes (95% CI)[3]**		
Less Acculturated, Lower SES	0.17(0.14,0.19)	0.20(0.12,0.33)	0.21(0.17,0.25)
Less Acculturated, Higher SES	0.14(0.12,0.17)	0.17(0.10,0.29)	0.18(0.14,0.23)
More Acculturated., Lower SES	0.14(0.11,0.16)	0.11(0.04,0.25)	0.18(0.15,0.22)
More Acculturated, Higher SES	0.11(0.09,0.13)	0.12(0.04,0.31)	0.15(0.12,0.18)
**Segmented Groups[2]**	**Incident Hypertension (95% CI)[3]**		
Less Acculturated, Lower SES	0.18(0.15,0.21)	0.16(0.09,0.25)	0.21(0.18,0.25)
Less Acculturated, Higher SES	0.15(0.12,0.18)	0.13(0.07,0.22)	0.18(0.15,0.22)
More Acculturated., Lower SES	0.19(0.15,0.23)	0.31(0.14,0.54)	0.21(0.17,0.25)
More Acculturated, Higher SES	0.10(0.08,0.12)	0.07(0.02,0.22)	0.12(0.10,0.15)

Note. CI = confidence interval, SES = socioeconomic status

[1]Analyses were weighted to account for complex sampling and study design of HCHS/SOL, age-standardized to the 2010 US Census population, and restricted to participants that completed Visit 1 and Visit 2 of HCHS/SOL.

[2]Acculturation level was dichotomized as low versus high based on the SASH-language subscale from 0-5, with values 0-1.9 categorized as low acculturation and values 2-5 categorized as high acculturation. SES was dichotomized as low versus high using educational attainment as a measure of socioeconomic status, with high school level or less categorized as low SES and some college or more as high SES.

[3]Regression models adjusted for age, gender, health insurance status, study site, family history of diabetes/hypertension respectively, obesity, poor diet, physical activity, and birthplace.

© 2025 Pérez-Stable EJ et al. *JAMA Health forum*.

eTable 5. Weighted predicted probabilities from modeling incident diabetes and incident hypertension at Visit 2 using variables at Visit 2, as a sensitivity analysis, in segmented groups stratified by heritage: Hispanic Community Health Study/Study on Latinos (n=11,623), ([2008-2011], [2014-2017])1

		Heritage				
	**Total** ***n*=11,623**	**Central American** ***n*=1,207**	**Cuban** ***n*=1,645**	**Dominican** ***n*=1,021**	**Mexican** ***n*=4,806**	**Puerto Rican** ***n*=1,801**
		**Incident Diabetes (95% CI)[3]**				
**Segmented Groups at visit 2[2]**						
Less Acculturated, Lower SES	0.20(0.18,0.24)	0.07(0.03,0.17)	0.16(0.10,0.26)	0.21(0.08,0.44)	0.21(0.13,0.32)	0.14(0.06,0.30)
Less Acculturated, Higher SES	0.18(0.14,0.22)	0.09(0.03,0.23)	0.16(0.09,0.27)	0.18(0.06,0.43)	0.17(0.10,0.28)	0.07(0.02,0.24)
More Acculturated., Lower SES	0.15(0.13,0.18)	0.02(0.01,0.08)	0.16(0.09,0.27)	0.04(0.01,0.18)	0.15(0.09,0.24)	0.12(0.05,0.26)
More Acculturated, Higher SES	0.11(0.09,0.14)	0.06(0.02,0.20)	0.07(0.04,0.13)	0.11(0.04,0.27)	0.11(0.06,0.18)	0.07(0.03,0.18)
**Age at visit 2, years**						
45	0.85(0.80,0.88)	0.87(0.70,0.95)	0.87(0.77,0.94)	0.83(0.59,0.94)	0.82(0.73,0.89)	0.89(0.79,0.95)
55	0.89(0.85,0.92)	0.91(0.74,0.97)	0.91(0.81,0.96)	0.88(0.62,0.97)	0.87(0.77,0.93)	0.93(0.83,0.97)
65	0.92(0.88,0.95)	0.94(0.78,0.99)	0.94(0.85,0.98)	0.91(0.64,0.98)	0.90(0.80,0.95)	0.95(0.87,0.98)
**Sex**						
Female	0.15(0.13,0.17)	0.05(0.02,0.13)	0.12(0.08,0.18)	0.14(0.06,0.31)	0.15(0.10,0.22)	0.08(0.03,0.19)
Male	0.17(0.15,0.20)	0.06(0.02,0.15)	0.15(0.10,0.22)	0.10(0.04,0.26)	0.16(0.10,0.25)	0.11(0.05,0.24)
**Health Insurance Status at visit 2**						
No insurance	0.15(0.12,0.17)	0.04(0.01,0.12)	0.13(0.09,0.20)	0.12(0.04,0.34)	0.14(0.09,0.22)	0.08(0.03,0.20)
Any insurance	0.17(0.15,0.19)	0.07(0.03,0.16)	0.14(0.09,0.21)	0.12(0.05,0.26)	0.17(0.11,0.25)	0.12(0.05,0.25)
**Center**						
Bronx	0.15(0.12,0.18)	0.05(0.02,0.13)	-	-	0.19(0.10,0.32)	0.06(0.03,0.13)
Chicago	0.18(0.16,0.21)	0.09(0.03,0.23)	-	-	0.15(0.10,0.22)	0.10(0.04,0.20)
Miami	0.11(0.10,0.13)	0.03(0.01,0.10)	-	-	0.12(0.03,0.33)	0.05(0.02,0.16)
San Diego	0.21(0.17,0.24)	0.06(0.02,0.19)	-	-	0.18(0.13,0.25)	0.25(0.04,0.72)
**Family history of diabetes**						
No	0.14(0.12,0.16)	0.04(0.02,0.12)	0.13(0.09,0.19)	0.09(0.03,0.25)	0.13(0.08,0.20)	0.09(0.04,0.21)
Yes	0.18(0.16,0.21)	0.07(0.03,0.16)	0.13(0.09,0.21)	0.15(0.07,0.32)	0.18(0.12,0.27)	0.11(0.05,0.22)
**Obese at visit 2 (BMI ≥30kg/m^2)**						
No	0.11(0.1,0.13)	0.03(0.01,0.07)	0.08(0.06,0.13)	0.09(0.03,0.20)	0.11(0.07,0.18)	0.07(0.03,0.16)
Yes	0.22(0.2,0.25)	0.11(0.05,0.24)	0.21(0.13,0.30)	0.16(0.06,0.37)	0.21(0.14,0.30)	0.14(0.06,0.28)
**Poor diet quality[4]**						
No	0.14(0.12,0.16)	0.05(0.02,0.12)	0.14(0.09,0.21)	0.12(0.05,0.27)	0.14(0.09,0.20)	0.07(0.03,0.15)
Yes	0.18(0.15,0.21)	0.06(0.02,0.17)	0.13(0.08,0.19)	0.12(0.03,0.36)	0.18(0.09,0.32)	0.14(0.06,0.30)
**Any physical activity[5]**						
Inactive	0.16(0.14,0.19)	0.04(0.01,0.12)	0.13(0.08,0.19)	0.15(0.05,0.38)	0.15(0.09,0.24)	0.09(0.04,0.21)
≥Low activity	0.16(0.14,0.18)	0.07(0.03,0.16)	0.14(0.09,0.21)	0.09(0.04,0.20)	0.16(0.10,0.24)	0.11(0.05,0.22)
**Nativity**						
Foreign Born	0.15(0.13,0.17)	0.08(0.04,0.13)	0.11(0.08,0.15)	0.10(0.04,0.21)	0.16(0.11,0.24)	0.05(0.01,0.19)
US/Territory Born	0.17(0.15,0.20)	0.04(0.01,0.19)	0.16(0.07,0.33)	0.15(0.03,0.48)	0.15(0.09,0.24)	0.19(0.11,0.31)
		**Incident Hypertension (95% CI)[3]**				
**Segmented Groups at visit 2[2]**						
Less Acculturated, Lower SES	0.18(0.15,0.21)	0.15(0.09,0.22)	0.24(0.13,0.41)	0.20(0.09,0.41)	0.09(0.05,0.15)	0.28(0.14,0.48)
Less Acculturated, Higher SES	0.14(0.11,0.17)	0.09(0.04,0.18)	0.22(0.11,0.37)	0.15(0.06,0.33)	0.06(0.03,0.10)	0.20(0.08,0.43)
More Acculturated., Lower SES	0.20(0.16,0.24)	0.15(0.07,0.30)	0.18(0.07,0.38)	0.23(0.09,0.46)	0.11(0.06,0.18)	0.22(0.1,0.41)
More Acculturated, Higher SES	0.11(0.08,0.13)	0.14(0.08,0.24)	0.13(0.06,0.25)	0.13(0.04,0.33)	0.05(0.03,0.09)	0.13(0.05,0.29)
**Age at visit 2, years**						

© 2025 Pérez-Stable EJ et al. *JAMA Health forum*.

eTable 5. Weighted predicted probabilities from modeling incident diabetes and incident hypertension at Visit 2 using variables at Visit 2, as a sensitivity analysis, in segmented groups stratified by heritage: Hispanic Community Health Study/Study on Latinos (n=11,623), ([2008-2011], [2014-2017])1

		Heritage				
	**Total** ***n*=11,623**	**Central American** ***n*=1,207**	**Cuban** ***n*=1,645**	**Dominican** ***n*=1,021**	**Mexican** ***n*=4,806**	**Puerto Rican** ***n*=1,801**
45	0.97(0.95,0.98)	0.99(0.96,0.99)	0.97(0.94,0.99)	0.99(0.96,0.99)	0.95(0.90,0.98)	0.91(0.76,0.97)
55	0.98(0.97,0.99)	0.99(0.98,0.99)	0.99(0.97,0.99)	0.99(0.98,0.99)	0.97(0.94,0.99)	0.94(0.80,0.99)
65	0.99(0.98,0.99)	0.99(0.99,0.99)	0.99(0.98,0.99)	0.99(0.99,0.99)	0.99(0.96,0.99)	0.97(0.84,0.99)
**Sex**						
Female	0.14(0.12,0.16)	0.12(0.07,0.20)	0.17(0.09,0.30)	0.13(0.06,0.26)	0.07(0.04,0.11)	0.21(0.10,0.38)
Male	0.16(0.14,0.19)	0.13(0.08,0.21)	0.21(0.11,0.36)	0.23(0.10,0.46)	0.08(0.05,0.13)	0.20(0.09,0.38)
**Health Insurance Status at visit 2**						
No insurance	0.14(0.11,0.16)	0.13(0.07,0.21)	0.21(0.11,0.35)	0.15(0.05,0.38)	0.06(0.03,0.11)	0.18(0.07,0.39)
Any insurance	0.17(0.15,0.19)	0.13(0.08,0.20)	0.17(0.09,0.31)	0.20(0.10,0.37)	0.09(0.06,0.14)	0.22(0.12,0.38)
**Center**						
Bronx	0.19(0.16,0.23)	0.13(0.07,0.22)	-	-	0.13(0.06,0.27)	0.30(0.17,0.49)
Chicago	0.13(0.11,0.16)	0.13(0.07,0.23)	-	-	0.07(0.04,0.12)	0.28(0.14,0.49)
Miami	0.18(0.15,0.21)	0.16(0.11,0.23)	-	-	0.05(0.01,0.24)	0.20(0.05,0.52)
San Diego	0.11(0.09,0.14)	0.10(0.03,0.26)	-	-	0.07(0.04,0.11)	0.09(0.03,0.25)
**Family history of hypertension**						
No	0.12(0.1,0.14)	0.10(0.06,0.16)	0.14(0.07,0.28)	0.13(0.05,0.28)	0.06(0.04,0.11)	0.18(0.08,0.34)
Yes	0.19(0.16,0.21)	0.16(0.10,0.25)	0.25(0.15,0.39)	0.23(0.10,0.45)	0.09(0.05,0.14)	0.23(0.11,0.42)
**Obese at visit 2 (BMI ≥30kg/m^2)**						
No	0.11(0.1,0.13)	0.09(0.05,0.15)	0.14(0.07,0.26)	0.11(0.05,0.25)	0.06(0.03,0.09)	0.15(0.07,0.30)
Yes	0.2(0.18,0.23)	0.18(0.11,0.27)	0.25(0.14,0.40)	0.26(0.11,0.48)	0.10(0.06,0.16)	0.27(0.13,0.46)
**Poor diet quality[4]**						
No	0.15(0.13,0.17)	0.11(0.08,0.16)	0.19(0.10,0.34)	0.13(0.06,0.25)	0.10(0.08,0.13)	0.22(0.10,0.40)
Yes	0.15(0.13,0.19)	0.14(0.07,0.27)	0.18(0.10,0.32)	0.23(0.08,0.52)	0.05(0.02,0.13)	0.19(0.08,0.38)
**Any physical activity[5]**						
Inactive	0.15(0.13,0.18)	0.14(0.08,0.24)	0.18(0.09,0.32)	0.20(0.08,0.42)	0.07(0.04,0.12)	0.24(0.11,0.44)
≥Low activity	0.15(0.13,0.17)	0.12(0.08,0.18)	0.19(0.11,0.33)	0.15(0.07,0.30)	0.08(0.05,0.13)	0.17(0.08,0.32)
**Birthplace**						
Foreign Born	0.15(0.13,0.17)	-	0.19(0.15,0.25)	0.22(0.12,0.37)	0.08(0.05,0.13)	0.24(0.07,0.57)
US/Territory Born	0.15(0.12,0.18)	-	0.18(0.05,0.48)	0.13(0.04,0.39)	0.07(0.04,0.12)	0.17(0.11,0.25)

Note. CI = confidence interval, SES = socioeconomic status

[1]Analyses were weighted to account for complex sampling and study design of HCHS/SOL, age-standardized to the 2010 US Census population, and restricted to participants that completed Visit 1 and Visit 2 of HCHS/SOL.

[2]Acculturation level was dichotomized as low versus high based on the SASH-language subscale of 0-5, with values 0-1.9 categorized as low acculturation and values of 2-5 categorized as high acculturation. SES was dichotomized as low versus high using educational attainment as a proxy measure of socioeconomic status, with high school level or less categorized as low SES and some college as high SES at Visit 2. If the Visit 2 value was missing, the Visit 1 value was carried forward.

[3]Regression models were adjusted for age at Visit 2, gender, health insurance status at Visit 2, study site, family history of diabetes/hypertension respectively, obesity at Visit 2, poor diet, physical activity, and birthplace (with the exception of models for (a) Cuban and Dominican heritage, which did not adjust for study site for incident diabetes and (b) Central American heritage, which did not adjust for birthplace for incident hypertension). If the Visit 2 value was missing, the Visit 1 value was carried forward.

[4]Poor diet quality was based on a score of <43 on the Alternative Healthy Eating Index 2010 (values ranged from 0 to 110).

[5]Inactive was based on no physical activity beyond those of basic living and greater than or equal to low activity was based on minutes of physical activity beyond those of basic living.

© 2025 Pérez-Stable EJ et al. *JAMA Health forum*.

eTable 6. Weighted predicted probabilities from modeling incident diabetes and incident hypertension at Visit 2 using change in control variables between Visit 1 and Visit 2, as a sensitivity analysis, in segmented groups stratified by heritage: Hispanic Community Health Study/Study on Latinos (n=11,623), ([2008-2011], [2014-2017])[1]

		Heritage				
	**Total** *n*=11,623	**Central American** *n*=1,207	**Cuban** *n*=1,645	**Dominican** *n*=1,021	**Mexican** *n*=4,806	**Puerto Rican** *n*=1,801
**Segmented Groups at Visit 2[2]**		**Incident Diabetes (95% CI)[3]**				
Less Acculturated, Lower SES	0.19(0.15,0.25)	0.05(0.01,0.16)	0.11(0.04,0.23)	0.11(0.03,0.33)	0.20(0.11,0.33)	0.13(0.04,0.34)
Less Acculturated, Higher SES	0.16(0.11,0.22)	0.08(0.02,0.27)	0.14(0.05,0.31)	0.19(0.04,0.58)	0.19(0.10,0.32)	0.02(0.00,0.08)
More Acculturated., Lower SES	0.12(0.09,0.16)	0.02(0.00,0.11)	0.10(0.03,0.27)	0.01(0.00,0.09)	0.10(0.05,0.19)	0.10(0.03,0.26)
More Acculturated, Higher SES	0.09(0.06,0.13)	0.08(0.02,0.31)	0.07(0.02,0.20)	0.14(0.04,0.44)	0.09(0.04,0.18)	0.03(0.01,0.11)
**Segmented Groups at Visit 2[2]**		**Incident Hypertension (95% CI)[3]**				
Less Acculturated, Lower SES	0.15(0.11,0.20)	0.14(0.07,0.28)	0.26(0.11,0.50)	0.26(0.08,0.59)	0.07(0.03,0.15)	0.17(0.06,0.39)
Less Acculturated, Higher SES	0.13(0.08,0.18)	0.09(0.03,0.28)	0.13(0.05,0.29)	0.08(0.01,0.34)	0.06(0.03,0.13)	0.25(0.07,0.62)
More Acculturated., Lower SES	0.17(0.12,0.23)	0.16(0.05,0.41)	0.20(0.06,0.50)	0.33(0.09,0.72)	0.07(0.03,0.16)	0.20(0.08,0.41)
More Acculturated, Higher SES	0.10(0.06,0.16)	0.14(0.05,0.34)	0.10(0.03,0.30)	0.09(0.01,0.46)	0.04(0.02,0.10)	0.23(0.07,0.53)

Note. CI = confidence interval, SES = socioeconomic status
[1]Analyses were weighted to account for complex sampling and study design of HCHS/SOL, age-standardized to the 2010 US Census population, and restricted to participants that completed Visit 1 and Visit 2 of HCHS/SOL.
[2]Acculturation level was dichotomized as low versus high based on the SASH-language subscale of 0-5, with values of 0-1.9 categorized as low acculturation and values of 2-5 categorized as high acculturation. SES was dichotomized as low versus high using educational attainment as a proxy measure of socioeconomic status, with high school level or less categorized as low SES and some college as high SES at Visit 2. If the Visit 2 value was missing, the Visit 1 value was carried forward.
[3]Regression models were adjusted for age at Visit 1, SASH language score at visit 1, change in age between visits, gender, health insurance status at Visit 1, change in health insurance status between visits, study site, family history of diabetes/hypertension respectively, BMI at Visit 1, change in BMI between visits, poor diet, physical activity, and birthplace (with the exception of (a) Cuban and Dominican heritage, which did not adjust for study site for incident diabetes and (b) Central American heritage, which did not adjust for birthplace for incident hypertension). If the Visit 2 value was missing, the Visit 1 value was carried forward.
[4]Poor diet quality was based on a score of <43 on the Alternative Healthy Eating Index 2010 (values from 0 to 110).
[5]Inactive was based on no physical activity beyond those of basic living and greater than or equal to low activity was based on minutes of physical activity beyond those of basic living.

© 2025 Pérez-Stable EJ et al. *JAMA Health forum*.
