# Supplementary material for: Diabetes and Hypertension Risk Across Acculturation and Education Levels in Hispanic/Latino Adults: The Hispanic Community Health Study/Study of Latinos
Source: JAMA Health Forum. 2025 Mar 28;6(3):e250273. doi: 10.1001/jamahealthforum.2025.0273 (PMC11953754; doi:10.1001/jamahealthforum.2025.0273)
Supplement: Supplement 2. — Data sharing statement [file jamahealthforum-e250273-s002.pdf]

Data Sharing Statement

Pérez-Stable. Diabetes and Hypertension Risk Across Acculturation and Education Levels in Hispanic/Latino Adults. *JAMA Health Forum*. Published March 28, 2025.
doi:10.1001/jamahealthforum.2025.0273

Data
Data available: Yes
Data types: Deidentified participant data
How to access data: Available from the Study Coordinating Center or NHLBI Intramural Program
When available: With publication

Supporting Documents
Document types: None

Additional Information
Who can access the data: Anyone requesting the data
Types of analyses: For research purposes
Mechanisms of data availability: After approval of a process
